# Mercy Hospital, Obstetrical Department

**Published:** 1879-08

**Authors:** H. M. Starkey

**Affiliations:** House Physician


### Clinical Reports.

### ARTICLE VI.

### MERCY HOSPITAL OBSTETRICAL DEPARTMENT.

Service by DR. ROLER.

Reported by H. M. STARKEY, M.D., House Physician.

*Pendent Uterus, Spontaneous Cranioclasm. Septicœmia. Double Phlegmasia Alba Dolens. Recovery.*

Julia F., age 22. Irish, domestic, medium size, well formed. Entered hospital Feb. 10, 1879, expecting to be confined in one week. Abdomen is noted as being unusually prominent, which prominence increased rapidly after the time patient expected to be confined.

Labor commenced in the evening of March 1, with very feeble pains. Patient says she has felt no life for nearly a week and thinks that the child is dead, but knows of no reason for this.

March 2, 2 p. m.—Pains are still very feeble. Neither the presenting part nor the os uteri can be touched per vaginam. By external manipulation, the head can be felt high up at the brim of the pelvis on the left side, while the breech is in the lower and dependent part of the abdomen on the right side. The placental bruit can be distinctly heard.

The head was crowded down into the pelvis, and the breech raised. This position of the fœtus was maintained by a binder placed firmly about the abdomen.

7 a. m.—Membranes ruptured and an unusually large amount of fluid escaped. Os about one-third dilated and occiput presenting. Abdomen still greatly distended.

No pains occurred after 10 a. m. during the day, and but one during the night.

March 3, 9 a. m.—Os about one-half dilated. There is an entire absence of labor pains, and we judge that the extreme distension of the uterus produced atony of that organ.

4 p. m.—A few weak pains occurred. The head had now descended to the vulva, and it is found that the child is dead, the head macerated and softened, the right parietal bone overriding the left, and the scalp ruptured. Soon the right parietal bone projected from the vulva and was withdrawn. The brains of the fœtus were removed, and by careful traction for about thirty minutes, the head was delivered. The pains were now entirely absent and a finger was hooked under the arm and considerable traction made in this way. By careful and patient labor in this manner for about forty minutes, the child was removed.

During these manipulations there were frequent discharges of very offensive gas, which passed away at times with such force, when some change in the position of the fœtus allowed an exit as to produce a loud noise similar to flatus escaping from the bowel. A large quantity also of decomposed and very offensive fluid escaped both during the expulsion of the child and after delivery.

The child was considerably decomposed, its abdomen distended with gas, and the umbilical cord nearly destroyed.

The uterus contracted well, expelled the placenta almost immediately, and there was very little hæmorrhage.

The patient's pulse was fairly strong and ninety-five per minute during labor, but soon after delivery her face became blanched and anxious, her respiration hurried and pulse soft, small and 120 per minute. Small and frequently repeated doses of brandy were directed, and quinine Gm. 2-6 every four hours alternately with fr. ferri. chlor. C.C. .5 in C.C. 4 of saturated solution potass. chloras.

Four hours after delivery temperature is 38.1°, pulse 120.

March 4, 8 a. m.—Temperature 38.1°, pulse 132. Complains of no pain. Medicines continued with addition of carbolized injections into the vagina.

March 5.—Temperature 39.5°, pulse 120 and stronger. Feeling a little less prostrated.

March 6.—Little change; temperature and pulse remaining high. Some tenderness over uterus, but no general abdominal tenderness and no tympanitis, but on the contrary abdominal walls are *very* lax, and can be gathered up in large rolls in the hands and have a pecular rough and leathery feel.

March 7, quinine replaced by

| | | |
|---|---|---|
| Tr. cinchonæ.............................. | 30 | C.C. |
| Spts. frumenti.......................... | 120 | |

Mix and give two teaspoonsful every four hours. Bowels obstinately constipated.

March 8.—Pulse, temperature and general appearance some better. Considerable swelling of labia minora, especially on right side. Laxatives and mild purgatives administered.

March 9.—Little change. Pulse ranges near 120 and temperature 39.2°–39.5°. Bowels only moved after repeated cathartics by mouth and by enema had been exhibited.

March 10.—Considerable improvement. This improvement continued steadily day by day till

March 15.—Temperature 38.4°, pulse 90–100. Sat up a few minutes while bed was made. Very little abdominal tenderness remaining. Complains of some soreness in calf of left leg.

March 16.—Endeavored to sit up, but pain and soreness of calf of left leg prevented. Leg is swelling a little.

March 17. Leg more swollen (though still moderately so) and pain very much increased. Marked tenderness along line of femoral vessels and their continuation down to ankle. Lump in left groin about size of walnut, and a tender swelling can be felt just over brim of pelvis. Applying to affected limb cloths saturated with carbolized zinc solution, as follows:

| | | |
|---|---|---|
| Acid. carbolicum.......................... | | 50 |
| Zinci chloridum............................. | | 20 |
| Aqua.............................................. | 30 | |

Mix.

Giving internally tr. ferri chlor C. C. | 5 in saturated solution of potassii chloralis C. C. | 4 every two hours. Temperature, 39°–39.5°.

March 18.—Temperature, 9 a.m., 38.8°. 9 p.m., 38.4°. Leg more swollen and swelling extends to abdomen. Pain very severe at times.

March 19, 9 a.m. Temp. 39°, pulse 120; 9 p.m., temp. 39.1°, pulse 120. Pain less severe, swelling more marked, especially along line of femoral vessels. Internal remedies continued. Locally, carbolized zinc lotion replaced by liniment consisting of

| | | |
|---|---|---|
| Spts. terebinthina...................... | | |
| Lin. sapon. camph. āā.................. | 25 | C. C. |
| Oleum olivum............................ | 50 | |

The leg is assuming the white and puffy appearance characteristic of phlegmasia alba dolens. March 20, 9 a.m. Temp. 38.7°, pulse 110; 9 p.m., temp. 39°, pulse 111. Leg continues to increase in size, but there is much less pain. The point of greatest tenderness is in the popliteal space, where there is a hardened cord a little to the inner side of the center.

March 21, 9 a.m. Temp. 38.1°, pulse 108. The circumferences of the left leg are: (a) Ankle over malleoli .248 M. (b) Ankle just above maleoli, .235 M. (c) Thickest part of calf, .362 M. (d) Knee, center of popliteal space and just below inferior border of patella, .343 M. (e) Centre of popliteal space and center of patella, .381 M. (f) .Middle of thigh, .495 M. Dimensions of sound limb at same time are: (a) .242 M, (b) .197 M, (c) .292 M, (d) .317 M, (e) .343 M, (f) .394 M.

March 22, 9 a.m; Temp. 37.65°, pulse 94. Limb almost entirely free from pain and soreness. The ankle is a little more swollen, but the dimensions of other parts of the limb are all less.

March 23, 9 a.m. Temp. 38°, pulse 100. Circumferences of left leg all diminished, and there is very little pain or tenderness. Some soreness remains in the groin, however. Patient complains of shooting pains through center of calf of right leg, and this point is found to be a little tender on pressure, and there is also a little stiffness of right knee; resembling suspiciously the commencement of the disease in the left leg.

March 24, 9 a.m. Temp. 38.5°, pulse 100. Improvement of left leg continues, but right leg shows more distinctly that it, also, is involved in the same disease. Ankle and calf swelling, very tender at calf and in groin, and very painful.

March 25, 9 a.m. Temperature, 38.3°, pulse 100. Left leg gives very little inconvenience, and dimensions are all the same as were noted two days ago. Right leg less painful, but swelling and tenderness are more marked. Has a milky white appearance, which is particularly noticeable about the ankle. Have for two days used the carbolized zinc solution mentioned before, but this is now changed for the turpentine liniment applied to the other leg.

March 26, 9 a.m. Temp. 38°, pulse 100. Suffers little pain in either leg unless moved, and there is marked decrease in the size of the left leg, except about the ankle, where there is more swelling, as will be seen by the following measurements :

*Left*—(a) .246 (b) .235 (c) .317 (d) .298 (e) .336 (f) .457
*Right*—(a) .235 (b) .215 (c) .292 (d) .305 (e) .343 (f) .419

March 27, 9 a.m. Temp. 38.6°, pulse 100. Little change; still using tr. ferri chlor. internally and turpentine liniment locally.

March 28, 9 a.m. Temp. 38.7°, pulse 98. Pain and tenderness of right leg much diminished, but is more swollen. Circumferences are :

*Left*—(a) .248 (b) .229 (c) .317 (d). 298 (e) .337 (f) .444

*Right*—(a) .260 (b) .229 (c) .317 (d) .305 (e) .343 (f) .419

March 31, 9 a.m. Temp. 37.1°, pulse 88. Improvement steady, swelling diminishing in all parts except right ankle, which is more swollen.

April 3, 9 a.m. Temp. 37°, pulse 86. Sits up in bed much of the time.

April 4. Fairly convalescent. Bore weight of body on left foot, but was unable to stand on right foot, and on attempting to do so fell on the floor. Circumferences to-day are :

*Left*—(a) .248 (b) .216 (c) .292 (d) .298 (e) .330 (f)) .419
*Right*—(a) .266 (b) .235 (c) .311 (d) .305 (e) .343 (f) .425

Left hospital nearly well.

Was seen April 28. Is sitting up all day, but walking is still a little difficult. Feet and ankles swell considerably in the daytime, but much of this disappears during the night. Was advised to wear an elastic bandage from the toes to the thighs.

Resumed work May 1.

